# Efficacy of aerobic physical retraining in a case of combined pulmonary fibrosis and emphysema syndrome: a case report

**DOI:** 10.1186/s13256-015-0570-3

**Published:** 2015-04-19

**Authors:** Giuseppe De Simone, Giovanna Aquino, Claudio Di Gioia, Gennaro Mazzarella, Andrea Bianco, Giuseppe Calcagno

**Affiliations:** 1 Department of Medicine and Health Sciences, “V Tiberio” University of Molise, Campobasso, Italy; 2 Institute of Rehabilitation “Villa Margherita Benevento”, Unit of Cardiology and Pneumology, Benevento, Italy; 3 Department of Cardio-Thoracic and Respiratory Sciences, Second University of Napoli, Napoli, Italy

**Keywords:** Aerobic physical retraining, Combined pulmonary fibrosis and emphysema, CPFE, Pulmonary rehabilitation

## Abstract

**Introduction:**

Combined pulmonary fibrosis and emphysema has recently been recognized as a syndrome but remains under-diagnosed. Neither clinical management nor therapeutic approaches have been clearly defined. Pulmonary rehabilitation has not been considered within the therapeutic options for combined pulmonary fibrosis and emphysema. In this case we explored the potential benefits of a specific aerobic physical retraining program in the management of combined pulmonary fibrosis and emphysema.

**Case presentation:**

We describe the case of a 65-year-old Caucasian man with combined pulmonary fibrosis and emphysema and respiratory failure who was receiving long-term oxygen therapy. Our patient underwent physical retraining with moderate intensity aerobic and breathing exercises for four weeks. Clinical and motor tests, as well as questionnaires assessing quality of life and depression levels, were performed prior to and following the retraining. At the end of the retraining program a relevant reduction of long-term oxygen therapy requirement was registered; improvements in terms of physical performance, quality of life, and mood were observed in our patient but no change in respiratory parameters.

**Conclusions:**

A program of aerobic physical retraining appears to be beneficial to patients with combined pulmonary fibrosis and emphysema and may be considered as an additional therapeutic option.

## Introduction

High-resolution computed tomography (HRCT) scanning has enhanced recognition of the simultaneous occurrence of emphysema and pulmonary fibrosis and, recently, combined pulmonary fibrosis and emphysema (CPFE) has been defined as a syndrome. This syndrome is characterized by upper lobe emphysema and lower lobe fibrosis [1]. Common risk factors resulting in epithelial lung alterations may contribute to emphysema and pulmonary fibrosis despite clinical, radiological, and pathologic differences [2-4]. Coexistence of emphysema and fibrosis are more likely to occur in smoking-induced parenchymal pulmonary damage [5,6]. CPFE is still under-diagnosed and clinical management as well as therapeutic approaches have not yet been clearly defined. In this case we explore the potential benefits of a specific aerobic physical retraining program, which has not yet been considered within the therapeutic options for CPFE.

## Case presentation

We report the case of a 65-year-old Caucasian man who had previously smoked cigarettes (40 pack-years) who had CPFE with several comorbidities, including hypertension, type II diabetes, and depression. Before his CPFE diagnosis our patient had been affected by chronic bronchitis for 12 years. His diagnosis of CPFE was confirmed by thoracic HRCT showing areas of intralobular interstitial and septal thickening with peripheral bilateral distribution in addition to centrilobular emphysema of both his upper lobes. On admission to our rehabilitation clinic, our patient exhibited chronic respiratory failure, reporting dyspnea on exertion and productive cough; our patient was receiving long-term oxygen therapy (LTOT) with 2.5L/min of flow during 24 hours as his peripheral capillary oxygen saturation (SpO_2_) steeply declined when oxygen therapy was discontinued (SpO_2_= 84%). Spirometry showed a relatively mild restriction although his diffusion lung capacity for carbon monoxide (DLCO) was 44% of predicted. Concurrent pulmonary hypertension (PAP 60mmHg) was diagnosed by color doppler echocardiography.

Our patient’s pharmacological therapy consisted of high dose inhaled steroids, acetyl-cysteine 1200mg once daily, angiotensin-converting-enzyme inhibitor (Lisinopril) 10mg once daily, and aspirin 100mg once daily. Respiratory and physical exercise test assessments were carried out at admission and discharge; in addition, quality of life (St. George Respiratory) and depression level (Geriatric Depression Scale) questionnaires were administered by a clinical psychologist. Our patient performed the six-minute walk test (6MWT) to evaluate his aerobic physical performance according to the European Respiratory Society/American Thoracic Society recommendation, 2014. The test was performed by an exercise physiologist indoors, along a long, flat, straight, enclosed corridor with a hard surface of 30 meters, and results reported as distance (meters) covered in six minutes. His usual medical regimen was continued and oxygen therapy during the test was administered via a portable oxygen tank. As required by standard protocol, our patient decided the speed of the walk, stopping as required and resuming the test when recovered. Before, during, and after the test, his heart rate and oxygen saturation were reordered using a professional oximeter, and dyspnea levels and overall fatigue were assessed according to the Borg Scale and Visual Analogue Scale (VAS) before and immediately after exercise test. After two weeks in our rehabilitation unit, our patient was clinically stabilized and enrolled in the retraining program for four weeks. The retraining program took place in the cardiorespiratory gymnasium of our rehabilitation unit. Written consent was obtained from our patient before starting the exercise program.

Each retraining session was supervised by an exercise physiologist monitoring heart and respiratory rate, oxygen saturation, blood pressure, and level of dyspnea. Our patient underwent the exercise program for two sessions per day up to 30 minutes each, five days per week, for a four-week period. The training included one session of aerobic exercise and one session of breathing technique. For aerobic training, he exercised on a treadmill, with a speed of 2.5km per hour and 0% of slope. The intensity of aerobic exercise was adapted to our patient’s functional capacity on the basis of his 6MWT results, in accordance with aerobic exercise intensity recommended by American Thoracic Society Guidelines 2006 for pulmonary rehabilitation. Therefore, the work rate of training corresponded to 50% to 60% of his maximum heart rate and the training began at a work rate equal to 50%. When our patient reached the level of exercise at 10 minutes without intolerable dyspnea (Borg rating of breathlessness of <5), the workload (speed and/or elevation) and duration of exercise were increased by 10% [7]. Progressive exercise training was employed for his respiratory muscles; the training consisted of diaphragmatic breathing and inspiratory muscle training, through an inspiratory threshold, at 40% to 50% of his initial maximal inspiratory pressure (PiMax). During training our patient received oxygen therapy to maintain SpO_2_ >92%; a flow reduction of 15% was made each week.

After the four-week retraining program, because his arterial blood gas analysis showed a PaO_2_ increase of 11% and PCO_2_ decrease of 5.6% on oxygen with a flow of 2.5L/min, it was possible to reduce his LTOT to a flow of 1.5L/min for 24 hours to compensate hypoxemia. The oxygen requirement reduction observed may have been due to the strengthening of his respiratory muscles, which allowed improvement of his exercise capacity [8]. His pulmonary artery systolic pressure, estimated by transthoracic two-dimensional echocardiography, improved by 16% in terms of cardiac index and pulmonary vascular resistance [9]; no significant lung function changes were observed. Relevant improvements in exercise capacity, dyspnea rating, health-related quality of life, and levels of depression were also demonstrated after the pulmonary rehabilitation program. His 6MWT showed an increase of distance walked by 133%. His dyspnea scores were reduced: the level of dyspnea at rest (the Modified Medical Research Council Dyspnea Scale) decreased from 3.0 to 2.0, Borg Scale during exercise was reduced from 9.0 to 5.0, and his post-exercise VAS decreased from 8.0 to 5.0; there was only a minor increase in PiMax of 17%. Our patient’s adherence to the retraining program was good and no adverse events occurred. Table 1 reports the test results collected before and after the retraining program.

**Table 1:** **Results of cardiorespiratory and clinical tests before and after a retraining program in a patient with combined pulmonary fibrosis and emphysema**

**Test**	**Assessment before retraining program**		**Assessment after retraining program**	
Respiratory functional tests	FEV1/FVC	**78%**	FEV1/FVC	**77%**
• Spirometry	FEV1	**79%** of predicted value	FEV1	**82%** of predicted value
	FVC	**73%** of predicted value	FVC	**75%** of predicted value
• Diffusion lung capacity for carbon monoxide	DLCO	**44%** of predicted value	DLCO	**47%** of predicted value
	PaO_2_	**63.4**mmHg	PaO_2_	**70.4**mmHg
Arterial blood gas analysis	PaCO_2_	**35.6**mmHg	PaCO_2_	**33.6**mmHg
on oxygen 2.5L/min	pH	**7.46**	pH	**7.369**
Dyspnea scales				
• Modified Medical Research Council		**3.0**		**2.0**
• Visual Analogue Scale		**8.0**		**5.0**
• Borg Scale		**9.0**		**5.0**
Transthoracic two-dimensional echocardiography	PAP	**60**mmHg	PAP	**50**mmHg
	Right ventricular hypertrophy		Right ventricular hypertrophy	
Physical exercise test				
• Respiratory muscle strength	PiMax	**40**mmHg	PiMax	**53**mmHg
	PeMax	**25**mmHg	PeMax	**60**mmHg
Six-minute walk test		**90**m^a^		**210**m
Geriatric Depression Scale		**25/30** severe depression		**16/30** mild depression
	Symptoms	**77.51**	Symptoms	**50.18**
Quality of life	Physical activities	**85.66**	Physical activities	**62.55**
St. George Questionnaire	Impact	**70.39**	Impact	**50.64**
	Total score	**83.86**	Total score	**71.54**
Long-term oxygen therapy	Flow 2.5L/min for 24 hours		Flow 1.5L/min for 24 hours	

^a^The six-minute walk test, before the retraining program, was stopped due to patient breathlessness. DLCO, diffusion lung capacity for carbon monoxide; FEV 1, Forced expiratory volume in one second; FVC, forced vital capacity; PaO_2_, partial arterial pressure of oxygen; PaCO_2_, partial pressure of carbon dioxide; PAP, pulmonary artery pressure; PiMax, maximal inspiratory pressure; PeMax, maximal expiratory pressure.

## Discussion

In this case study we evaluated whether a patient with CPFE, a disease which has not yet been considered for pulmonary rehabilitation, can benefit from an aerobic retraining program. Emphysema and pulmonary fibrosis are progressive lung diseases that may be associated with comorbidities and systemic consequences [10-14]. CPFE syndrome typically occurs in male smokers and is frequently complicated by pulmonary hypertension, acute lung injury, and lung cancer, which affect disease progression and survival. In the literature there are no reports on the efficacy of physical retraining in patients with CPFE, in contrast with other lung diseases such as chronic obstructive pulmonary disease or idiopathic pulmonary fibrosis where clinical studies have demonstrated the benefits of pulmonary rehabilitation [15]. Clinically relevant improvements and short-term benefits were clearly demonstrated in this report as a result of a retraining program. Although clinical studies on long-term effects within a large patient population are required for definitive conclusions, our observations suggest that integrating pulmonary rehabilitation with pharmacological treatments would be beneficial for patients with CPFE.

## Conclusions

In the literature there are no reports on the effects of pulmonary rehabilitation in patients with CPFE. The potential efficacy of aerobic exercise on quality of life and psychological well-being and exercise capacity function in a patient with CPFE has been demonstrated in this case. Our results suggest that exercise should also be considered as a therapeutic option for patients with CPFE, although further studies considering the impact of different types of training in this patient population are required.

## Consent

Written informed consent was obtained from the patient for publication of this case report. A copy of the written consent is available for review by the Editor-in-Chief of this journal.

## Abbreviations

DLCO: diffusion lung capacity for carbon monoxide
FEV 1: Forced Expiratory Volume in one second
FVC: Forced Vital Capacity
HRCT: high-resolution computed tomography
LTOT: long-term oxygen therapy
PaO_2_: partial arterial pressure of oxygen
PaCO_2_: partial pressure of carbon dioxide
PAP: pulmonary artery pressure
PiMax: maximal inspiratory pressure
PeMax: maximal expiratory pressure
6MWT: six-minute walk test, VAS, Visual Analogue Scale
